# Supplementary figures and images for: An Open Source Platform for Presenting Dynamic Visual Stimuli
Source: eNeuro. 2021 Jun 10;8(3):ENEURO.0563-20.2021. doi: 10.1523/ENEURO.0563-20.2021 (PMC8205497; doi:10.1523/ENEURO.0563-20.2021)

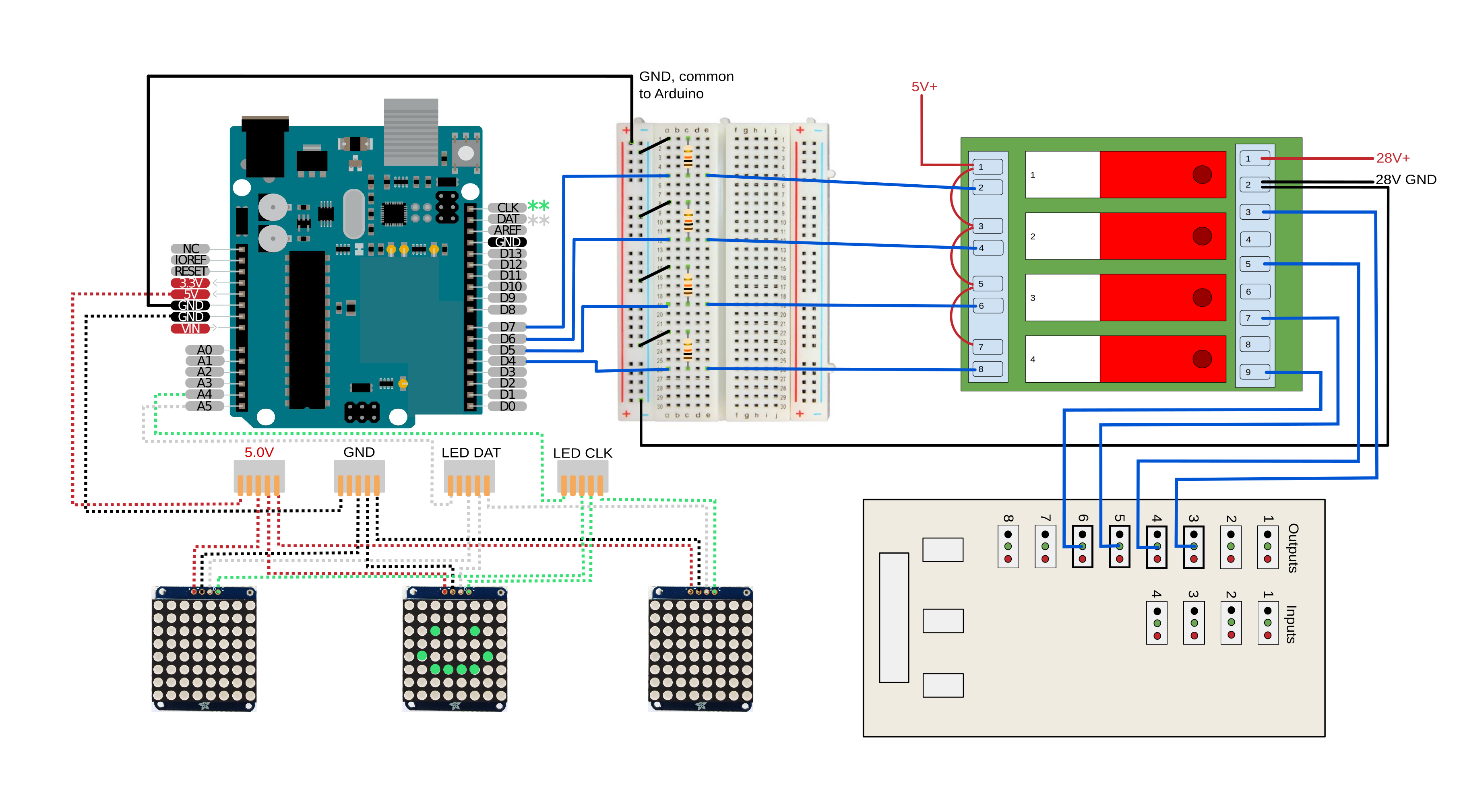

Supplement: Extended Data 1 — • README.txt, overview on files in the Extended Data 1. • StimCodes.png, documentation on producing the visual patterns over three LED matrices as described in this manuscript. • LEDMatrix-Addressing.png, documentation on addressing for the LED Backpacks. • WiringDiagram.png, image showing specific wiring for the LED Backpacks, Arduno, and breadboard. • FullSetUp.png, image showing wiring diagram for LED Backpacks, Arduino, breadboard, and relays to integrate with MedPC control system. • nosepoke.stl and nosepoke-block.stl, design files for the optional nosepoke ports. • LED-Matrix-Holder-Part1.stl and LED-Matrix-Holder-Part2.stl, design files for the 3D-printed holders for the LED matrices. • VisualStimuli-Arduino-eNeuro.ino, Arduino code for the device. The following files are included in the Extended Data, which can be found at https://github.com/LaubachLab/LED-matrices: Download Extended Data 1, ZIP file. [file enu-eN-OTM-0563-20-s01.zip › LED-Matrices-eNeuro-ExtendedData/FullSetUp.png]

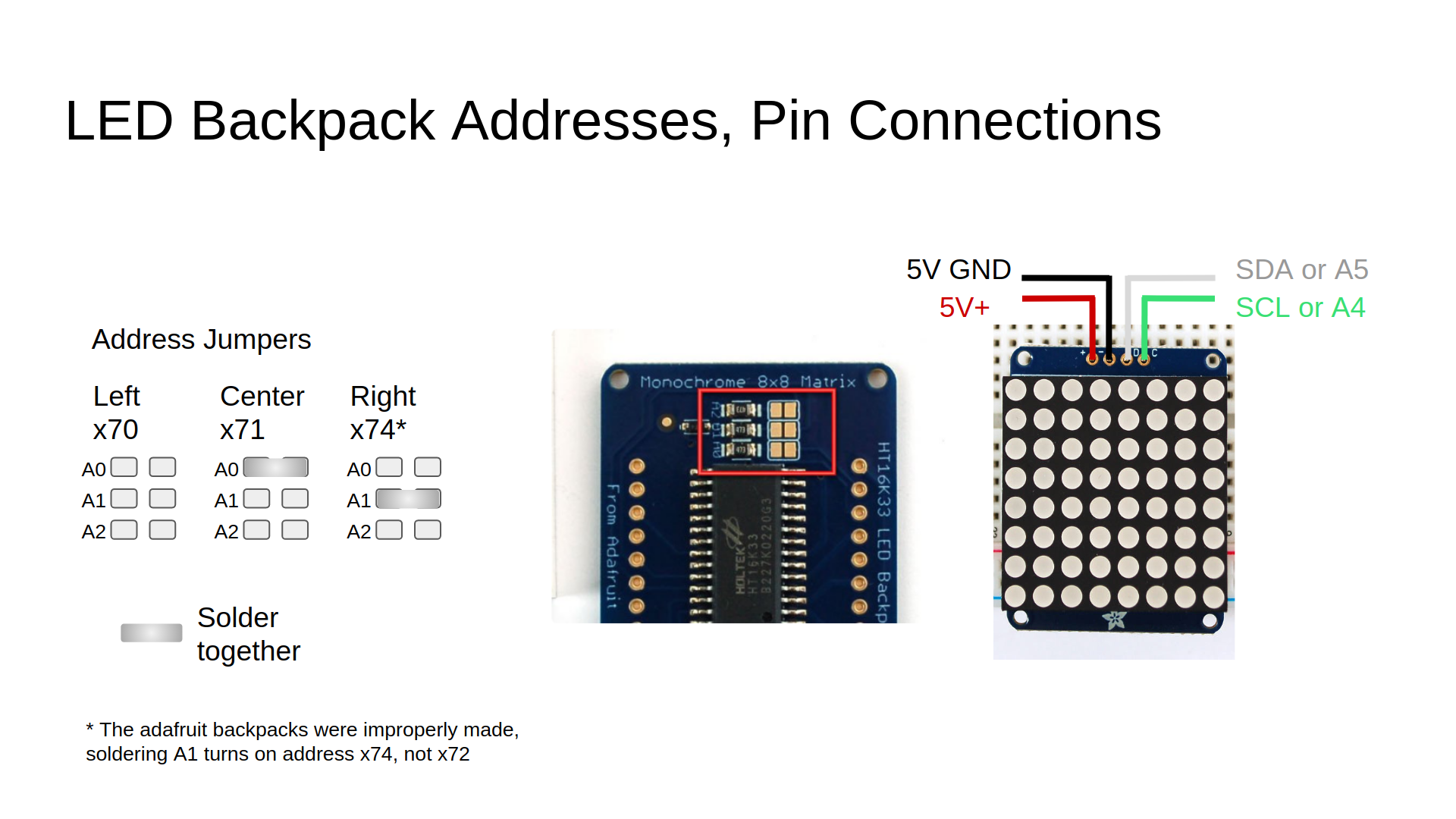

Supplement: Extended Data 1 — • README.txt, overview on files in the Extended Data 1. • StimCodes.png, documentation on producing the visual patterns over three LED matrices as described in this manuscript. • LEDMatrix-Addressing.png, documentation on addressing for the LED Backpacks. • WiringDiagram.png, image showing specific wiring for the LED Backpacks, Arduno, and breadboard. • FullSetUp.png, image showing wiring diagram for LED Backpacks, Arduino, breadboard, and relays to integrate with MedPC control system. • nosepoke.stl and nosepoke-block.stl, design files for the optional nosepoke ports. • LED-Matrix-Holder-Part1.stl and LED-Matrix-Holder-Part2.stl, design files for the 3D-printed holders for the LED matrices. • VisualStimuli-Arduino-eNeuro.ino, Arduino code for the device. The following files are included in the Extended Data, which can be found at https://github.com/LaubachLab/LED-matrices: Download Extended Data 1, ZIP file. [file enu-eN-OTM-0563-20-s01.zip › LED-Matrices-eNeuro-ExtendedData/LEDMatrix-Addressing.png]

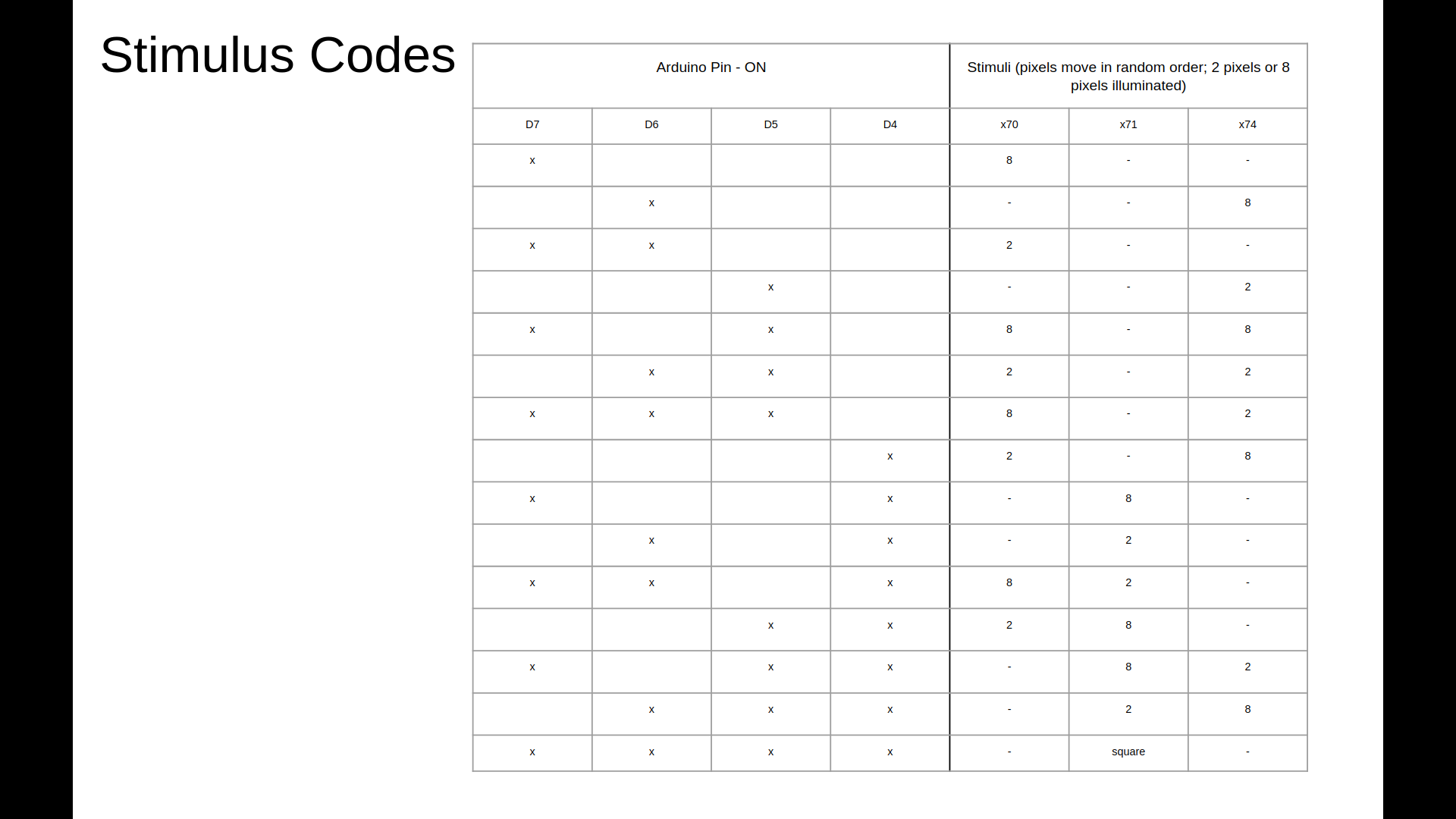

Supplement: Extended Data 1 — • README.txt, overview on files in the Extended Data 1. • StimCodes.png, documentation on producing the visual patterns over three LED matrices as described in this manuscript. • LEDMatrix-Addressing.png, documentation on addressing for the LED Backpacks. • WiringDiagram.png, image showing specific wiring for the LED Backpacks, Arduno, and breadboard. • FullSetUp.png, image showing wiring diagram for LED Backpacks, Arduino, breadboard, and relays to integrate with MedPC control system. • nosepoke.stl and nosepoke-block.stl, design files for the optional nosepoke ports. • LED-Matrix-Holder-Part1.stl and LED-Matrix-Holder-Part2.stl, design files for the 3D-printed holders for the LED matrices. • VisualStimuli-Arduino-eNeuro.ino, Arduino code for the device. The following files are included in the Extended Data, which can be found at https://github.com/LaubachLab/LED-matrices: Download Extended Data 1, ZIP file. [file enu-eN-OTM-0563-20-s01.zip › LED-Matrices-eNeuro-ExtendedData/StimCodes.png]

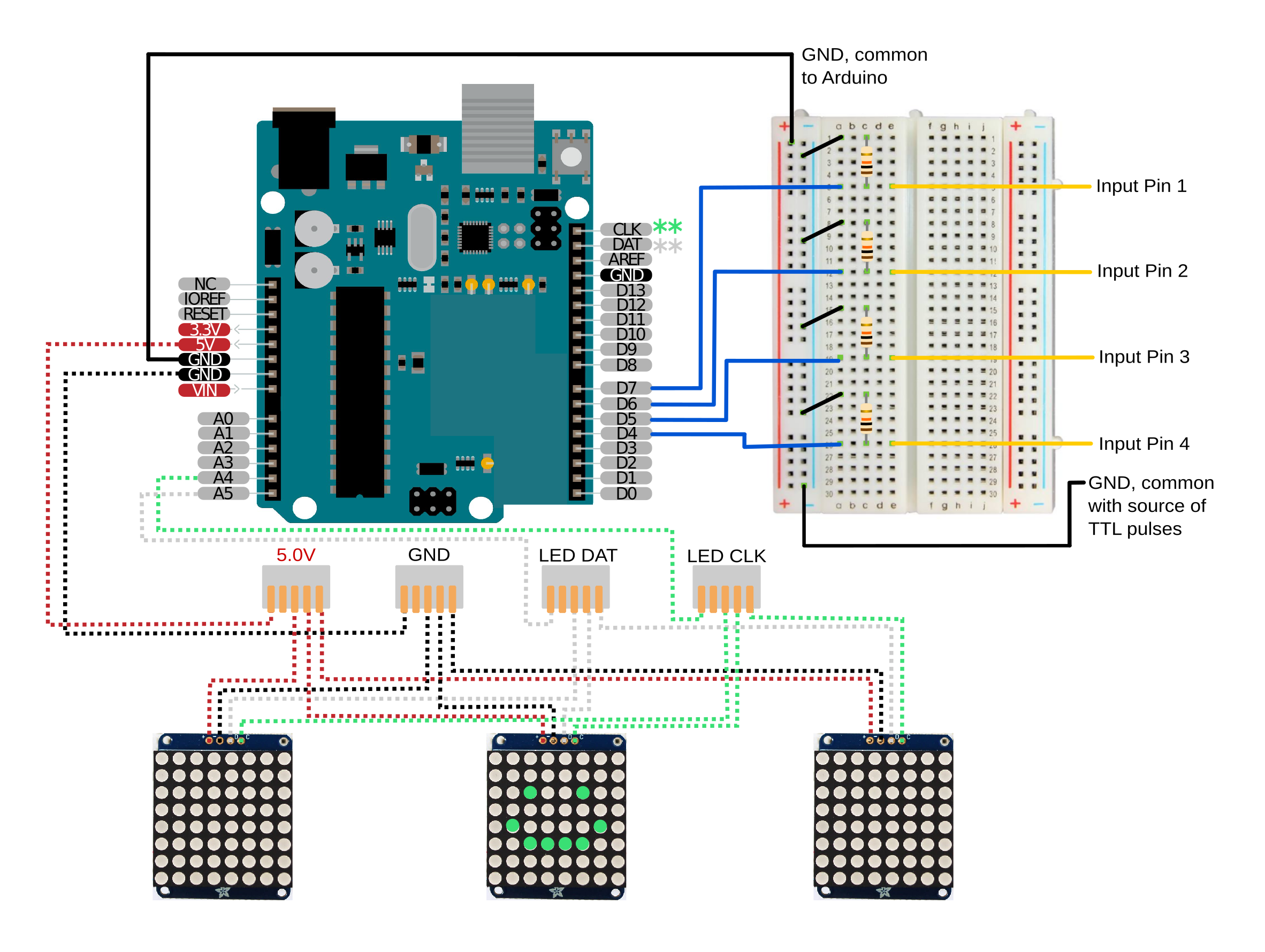

Supplement: Extended Data 1 — • README.txt, overview on files in the Extended Data 1. • StimCodes.png, documentation on producing the visual patterns over three LED matrices as described in this manuscript. • LEDMatrix-Addressing.png, documentation on addressing for the LED Backpacks. • WiringDiagram.png, image showing specific wiring for the LED Backpacks, Arduno, and breadboard. • FullSetUp.png, image showing wiring diagram for LED Backpacks, Arduino, breadboard, and relays to integrate with MedPC control system. • nosepoke.stl and nosepoke-block.stl, design files for the optional nosepoke ports. • LED-Matrix-Holder-Part1.stl and LED-Matrix-Holder-Part2.stl, design files for the 3D-printed holders for the LED matrices. • VisualStimuli-Arduino-eNeuro.ino, Arduino code for the device. The following files are included in the Extended Data, which can be found at https://github.com/LaubachLab/LED-matrices: Download Extended Data 1, ZIP file. [file enu-eN-OTM-0563-20-s01.zip › LED-Matrices-eNeuro-ExtendedData/wiringDiagram.png]
